# Supplementary material for: Is Childhood Overweight/Obesity Perceived as a Health Problem by Mothers of Preschool Aged Children in Bangladesh? A Community Level Cross-Sectional Study
Source: Int J Environ Res Public Health. 2019 Jan 12;16(2):202. doi: 10.3390/ijerph16020202 (PMC6352241; doi:10.3390/ijerph16020202)
Supplement: Supplementary file 1 [file ijerph-16-00202-s001.pdf]

Supplementary Materials: Is Childhood Overweight/Obesity Perceived as a Health Problem by Mothers of Preschool Aged Children in Bangladesh? A Community Level Cross-Sectional Study

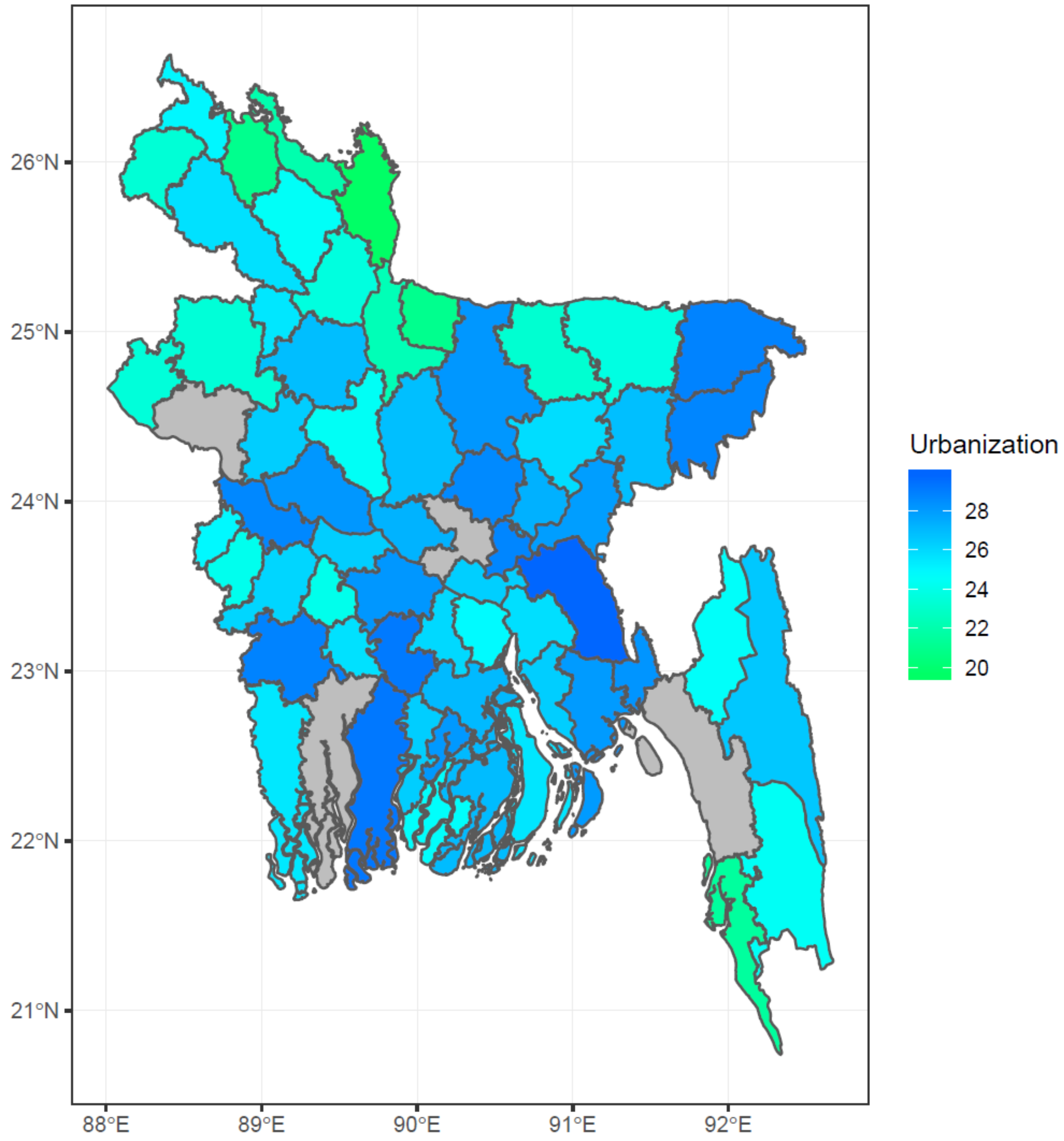

Figure S1. District town specific percentage of urbanisation based on a calculated urbanisation index utilizing data on population size, literacy rate, percentage of population with access to tap water for drinking, percentage with sanitary toilet facilities, and percentage with access to electricity.

Table S1. Perceptions and knowledge of childhood overweight and obesity by preschool type.

Variable	Category	Secular (%, *n*)	Noorani (%, *n*)	*p*-Value
Childhood obesity is a health problem	Yes	36.1 (142)	33.5 (63)	0.81
	No	53.2 (209)	55.9 (105)	
	Don't know	10.7 (42)	3.4 (20)	
Childhood obesity is a sign of good health	Yes	26.0 (102)	23.4 (44)	0.70
	No	34.7 (136)	34.0 (64)	
	Don't Know	39.3 (154)	42.6 (80)	
An obese child will be healthy when becomes adult	Yes	11.2 (44)	8.5 (16)	0.13
	No	10.2 (40)	15.4 (29)	
	Don't Know	78.6 (309)	76.1 (143)	
Consuming junk food	Yes	41.0 (161)	33.5 (63)	0.07
	No	22.9 (90)	20.7 (39)	
	Don't Know	36.1 (142)	45.7 (86)	
Lack of physical activity	Yes	39.9 (157)	35.6 (67)	0.57
	No	18.8 (74)	19.1 (36)	
	Don't Know	41.2 (162)	45.2 (85)	
Spending more than 2 h screen time	Yes	29.3 (115)	30.9 (58)	0.007
	No	30.5 (120)	18.6 (35)	
	Don't Know	40.2 (158)	50.5 (95)	
Lack of play ground	Yes	44.0 (173)	43.3 (81)	0.04
	No	21.6 (85)	13.98 (26)	
	Don't Know	34.4 (135)	42.8 (80)	
Knowledge of any health consequences of childhood obesity	Yes (Any other)	23.4 (92)	25.0 (47)	0.73
	Don't Know	76.6 (302)	75.0 (141)	

Table S2: Proportion of perception (Yes)

Variables	Sex		Mother's Education		Father's Education		Monthly Income		Child's Status		
	**Boy** %, *n*	**girl** %, *n*	**PS** %, *n*	**HG** %, *n*	**PS** %, *n*	**HG** %, *n*	**<10K** %, *n*	**25–50+K** %, *n*	**Underweight** %, *n*	**Normal** %, *n*	**Overweight/Obese** %, *n*
Childhood obesity is a health problem	62.4 (128)	37.6 (77)	42.6 (87)	57.4 (117)	32.8 (65)	67.2 (133)	42.2 (62)	57.8 (85)	29.8 (61)	55.1 (113)	15.1 (31)
Childhood obesity is a sign of good health	55.5 (81)	44.5 (65)	47.6 (69)	52.4 (76)	36.4 (52)	63.6 (91)	54.6 (59)	45.4 (49)	31 (45)	60 (87)	9 (13)
An obese child will be healthy when becomes adult	60 (36)	40 (24)	60 (36)	40 (24)	55.0 (33)	45.0 (27)	70.5 (31)	29.5 (13)	18.6 (11)	71.2 (42)	10.2 (6)
Family member will be critical if child is thin	54 (141)	46 (120)	54.4 (142)	45.6 (119)	43.8 (113)	56.2 (145)	58.1 (111)	41.9 (80)	38.2 (99)	52.9 (137)	8.9 (23)
Factors contributing childhood obesity											
Consuming junk food	54.5 (122)	45.5 (102)	31.5 (70)	68.5 (152)	22.8 (50)	77.2 (169)	30 (48)	70 (112)	25.6 (57)	53.8 (120)	20.6 (46)
Lack of physical activity	57.1 (128)	42.9 (96)	29.5 (66)	70.5 (158)	21.2 (47)	78.8 (175)	34.4 (55)	65.6 (105)	25.1 (56)	55.2 (123)	19.7 (44)
Spending more than 2 h screen time	61.3 (106)	38.7 (67)	37.6 (65)	62.4 (108)	27.9 (48)	72.1 (124)	39.1 (50)	60.9 (78)	27.3 (47)	51.2 (88)	21.5 (37)
Lack of play ground	57.1 (145)	42.9 (109)	37.4 (95)	62.6 (159)	25.8 (65)	74.2 (187)	37.3 (69)	62.7 (116)	28.1 (71)	56.1 (142)	15.8 (40)
